# Schwartz rounds for healthcare personnel in coping with COVID-19 pandemic

**DOI:** 10.1136/postgradmedj-2020-137806

**Published:** 2020-04-28

**Authors:** Jeyasakthy Saniasiaya, Kuganathan Ramasamy

**Affiliations:** Department of Otorhinolaryngology, Hospital Tuanku Ja'afar, Seremban, Negeri Sembilan, Malaysia; Department of Otorhinolaryngology, Hospital Tuanku Ja'afar, Seremban, Negeri Sembilan, Malaysia

**Keywords:** education & training (see medical education & training), quality in health care

The world watched in apathy as China struggled with rising numbers of the coronavirus, which took place in Wuhan, China, in early December 2019.^1^ Little did they know how an innocent encounter with a bat would bring the world to its knees, leading to a global pandemic in just 2 months. An alarming level of concern emerged when cases outside China revealed an appalling 13-fold rise in less than 2 weeks, urging the pandemic siren by WHO.

This became the biggest challenge for the healthcare personnel (HCP), who instantaneously were forced to become superheroes. For most HCPs, waking up daily to an alarming number of death tolls and infected cases, overwhelming workload, lack of personal protective equipment, as well as lack of treatment have surpassed the concern of general well-being. Day-to-day yearning to come home safely to their loved ones puts every HCP in a state of anxiety. Interestingly, we are beginning to realise how high school home science lessons have finally become handy, turning HCP into craftsmen and seamstresses to manufacture in-house personal protective equipment as it becomes a dire shortage. Setting off to duty day after day with fear and trepidation takes a toll physically and mentally. All eyes and effort in curbing the pandemic, with least attention paid to the psychosocial effect on HCPs, may lead to a detrimental effect.

We would like to propose Schwartz rounds (SRs) to be a part of daily routine in all healthcare facilities during this COVID-19 pandemic. ‘The smallest act of kindness made the unbearable bearable’ were the words written by Kenneth Schwartz in *The Boston Globe*.^2^ Kenneth Schwartz, a successful American lawyer who succumbed 11 months after being diagnosed with lung cancer at Brigham and Women’s Hospital, Boston, pioneered SR following the outstanding compassionate care he received during his entire hospitalisation period. SR was introduced to allow HCPs encompassing the entire hospital staff, not only involving doctors and nurses but also porters, catering staff, pharmacists, librarians, managers and administrators, to freely express on the care of a particular patient. This later bloomed across the globe and became a platform for HCPs to express their emotional, social well-being, as well as ethical challenges faced at work.^3^

Schwartz Center for Compassionate Healthcare was developed in 1995 in the USA. In the UK alone, SRs are practised in over 170 UK health and social care organisations, including hospitals, hospices, as well as community settings. Participating organisations have trained facilitators worldwide on how SR can be conducted while providing continuous support. Additionally, myriad publications have reported on the benefits of SR to HCPs, including stabilising emotions, reducing isolation, providing support and compassionate care, as well as improvement in patient care, which will be beneficial during this ‘dark period’. SR demonstrated immense improvement in patient compassion, better teamwork, as well as improvement in psychosocial well-being among staff, which enhances performance in organisations. Unlike the selective group of HCPs which incorporates mental health issues, such as mental health nurses, midwives, psychologists and social workers; most doctors and nurses have little or no support with psychosocial well-being. It is during this unprecedential time that the practise of SR is needed the most. It is saddening when psychological counselling for HCPs is stigmatised.

Question may arise as to when is the right time to conduct SR in the healthcare facility despite the burgeoning cases of COVID-19. Traditional SR proposed by Kenneth Schwartz himself begins with a buffet lunch followed by a 1-hour meeting. As time is not on our side, a short interval of discussion can be carried out during the changing shift period in healthcare. Head of management, chair of departments, team leaders or staff leaders ought to conduct discussion periodically to ensure the well-being of all HCPs is maintained.

‘Moral injury’ resulting from action or inaction which violates moral or ethical code, causing psychological trauma, may take a toll on HCPs. The recently disheartening news on Italian physicians who were devastated as well as abashed, as they were forced to decide on which patients need ventilators the most,^4^ startled humanity. This is among the moral injuries which entail SR as these groups of guilt-ridden HCPs not only may suffer in the long run from depression but also may find it hard to continue their journey in this pandemic period. A recent survey study by Lai *et al* involving HCPs directly dealing with patients with COVID-19 in China revealed high rates of depression, anxiety, insomnia and distress.^5^ It is also noteworthy that frontline HCPs handling these patients, nurses and women, as well as those working in Wuhan, showed the worst mental health outcome.^5^

It is imperative to take note that the worst is yet to come as we also need to think on the postacute crisis following this deadly pandemic, which may be more demanding to HCPs. The hardest battle is to win against oneself; thus, it behoves HCPs for the implementation of early mental healthcare measures such as Schwartz rounds since HCPs stand as the most crucial team in curbing this novel pandemic.
